# Cardiac Sarcoidosis and Inherited Cardiomyopathies: Clinical Masquerade or Overlap?

**DOI:** 10.3390/jcm14051609

**Published:** 2025-02-27

**Authors:** Sami Fouda, Rebecca Godfrey, Christopher Pavitt, Thomas Alway, Steven Coombs, Susan M. Ellery, Victoria Parish, John Silberbauer, Alexander Liu

**Affiliations:** 1 West Middlesex Hospital, London TW7 6AF, UK; sami.fouda1@nhs.net; 2 Sussex Cardiac Centre, Royal Sussex County Hospital, Brighton BN2 5BE, UK; rebecca.godfrey@nhs.net (R.G.); christopher.pavitt@nhs.net (C.P.); t.alway@nhs.net (T.A.); steven.coombs@nhs.net (S.C.); susan.ellery1@nhs.net (S.M.E.); victoria.parish@nhs.net (V.P.); john.silberbauer@nhs.net (J.S.)

**Keywords:** cardiac sarcoidosis, inherited cardiomyopathies, arrhythmogenic cardiomyopathy, hypertrophic cardiomyopathy, dilated cardiomyopathy, clinical overlap

## Abstract

Cardiac sarcoidosis (CS) and inherited cardiomyopathies (inherited CM) are associated with advanced heart failure, cardiac conduction defects, ventricular arrhythmias and sudden cardiac death. Both conditions can have similar clinical presentations. Differentiating between the two disease cohorts is important in delivering specific management to patients, such as immunosuppressive therapy for CS patients and genetic screening for inherited CM. In this review, we examined the existing evidence on the overlap between CS and common inherited CM, such as hypertrophic cardiomyopathy, arrhythmogenic cardiomyopathy, restrictive cardiomyopathy and dilated cardiomyopathy. In patients where both CS and inherited CM were implicated, CS tended to be diagnosed much later, often when patients presented with complications warranting a workup or cardiac histological confirmation. CS can masquerade as an inherited CM, leading to delays in the instigation of CS therapy. Confirmed dual pathology overlap between inherited CM and CS is rarer. Advanced cardiac imaging, such as cardiovascular magnetic resonance, plays an important role in the clinical workup of both CS and inherited CM. However, findings on cardiac imaging alone often cannot differentiate between the two conditions. Definitive differentiation between CS and inherited CM requires both clinical experience and, at times, a myocardial biopsy.

## 1. Introduction

Sarcoidosis is a chronic multi-system inflammatory condition characterised by the presence of non-caseating granulomas in affected tissues [1]. The prevalence of sarcoidosis varies from up to 160 per 100,000 in Scandinavian countries and much less in East Asian countries [1]. Cardiac involvement occurs in around a quarter of sarcoidosis patients, who may present with heart failure, high-grade atrioventricular heart block (AVB), ventricular arrhythmias and/or sudden cardiac death (SCD) [1]. Early diagnosis of patients with cardiac sarcoidosis (CS) enables early instigation of treatments, which may alter the long-term prognosis [1,2]. The diagnosis of CS can be challenging since no single test provides comprehensive diagnostic performance for the condition [2]. Although the finding of non-caseating granulomas on endomyocardial biopsy is considered a gold-standard diagnosis of CS, cardiac tissue sampling is prone to false negatives due to the patchy nature of CS [2]. In practice, careful review of the clinical information and advanced cardiac imaging data is important in the diagnostic and management processes [1,2,3,4].

Inherited cardiomyopathies (inherited CM) constitute a heterogeneous range of cardiomyopathies often associated with genetic variants, such as those coding for cardiomyocyte contractile proteins and desmosomes [5]. Well-characterised inherited CMs include hypertrophic cardiomyopathy (HCM, e.g., MYH7 gene mutation; OMIM CMH), dilated cardiomyopathy (DCM, e.g., SGCD gene mutation; OMIM CMD), and arrhythmogenic cardiomyopathy (ACM, e.g., LMNA gene mutation), for which established clinical pathways are available for diagnosis and management [5]. Patients with inherited CM can present at a young age with potentially serious complications such as heart failure, advanced AVB, ventricular arrhythmias and SCD [5]. The potential heritable nature of inherited CM means that a timely diagnosis has clinical ramifications for both the patients and their relatives [5]. Over the last two to three decades, increased awareness of inherited CM and advances in diagnostic and therapeutic methods have enabled the development of well-established services catered for the clinical needs of inherited CM patients [6]. Whilst inherited CM diagnosed in adults has received significant clinical attention, inherited CM can also lead to morbidity and mortality in children, with an approximate incidence of 1.1–1.5 per 100,000 in young people below the age of 18 years. Dilated cardiomyopathy (DCM) and hypertrophic cardiomyopathy (HCM) constitute the majority of paediatric inherited CM [7].

Differentiating between CS and inherited CM can be challenging from the outset, constituting an important clinical conundrum [8]. CS may mimic inherited CM in clinical presentation and advanced cardiac imaging findings [9,10,11,12,13,14]. There is also some evidence suggesting that CS and inherited CM can co-exist in the same patient, which adds to the complexity of making a clinical diagnosis [8,15]. While some aspects of clinical management are similar between the two disease cohorts, such as managing heart failure and SCD risk, significant differences exist [1,5]. Whilst detecting and treating myocardial inflammation is an important part of CS patient management, genetic testing and other specific therapies, such as left ventricular outflow tract obstruction (LVOTO) management for HCM, are much more relevant in the effective care of inherited CM patients [1,5]. Indeed, treating CS patients as if they had inherited CM, and vice versa, may be detrimental. It is, therefore, important that clinicians have a firm understanding of the potential overlap between CS and inherited CM to support heightened clinical vigilance when managing these patients. This review will present evidence of the possible overlap and/or masquerade between CS and inherited CM.

## 2. Clinical Symptoms and Presentation

There are several similarities between CS and inherited CM in regard to symptomology, which presents a diagnostic challenge in the initial clinical assessment [1,5]. Patients with either condition can be asymptomatic, and the disease may be first detected on screening. For CS, this tends to be in patients with extra-cardiac sarcoidosis who present with an abnormal ECG and/or cardiac dysfunction on echocardiography, which can trigger the performance of workup with advanced cardiac imaging, including cardiovascular magnetic resonance (CMR) and 18F-fluorodeoxyglucose (FDG) positron emission tomography/computed tomography (PET/CT) [1,3]. For inherited CM, patients can be diagnosed after screening of asymptomatic patients, for instance, with cascade screening of first-degree relatives of a proband [5].

In clinically manifest patients, CS and inherited CM may present with similar cardiac symptoms (e.g., chest discomfort, breathlessness, palpitations and/or syncope) [1,5]. These symptoms tend to be non-specific in differentiating between CS and inherited CM, and they may represent underlying sequelae such as arrhythmias, heart failure and advanced atrioventricular block (AVB), which can occur in both disease cohorts [1,5]. In both conditions, cardiac arrest and sudden cardiac death can happen but are relatively rare [1,5].

Although the development of cardiac symptoms in patients with known extra-cardiac sarcoidosis should trigger screening and workup for CS, symptom development itself does not differentiate between CS and inherited CM [1,5]. Whilst suggestive findings on advanced cardiac imaging may increase the likelihood of a CS diagnosis being reached in patients with systemic sarcoidosis, these findings alone make it often difficult to differentiate between CS and inherited CM [8,11,16,17].

## 3. Diagnostic Modalities in CS and Inherited CM

### 3.1. Transthoracic Echocardiography (TTE)

TTE is the first-line non-invasive imaging test in the workup for CS and inherited CM [1,5]. Despite its high specificity (95–98%), its sensitivity for detecting CS tends to be low (25–27%), particularly in early disease [18,19,20]. Specific TTE findings, such as left ventricular (LV) hypertrophy, LV and RV dilatation, dysfunction and reduced global longitudinal strain, can be present in both CS and inherited CM [1,5]. However, these features in clinical practice may raise clinical suspicion for inherited CM more than CS in the absence of other suggestive features for sarcoidosis [20,21,22]. TTE may uncover features more suggestive of CS, such as basal interventricular septum (IVS) thinning [1] and/or regional wall motion abnormalities (RWMA) in a non-coronary distribution [23,24]. The presence of hyper-trabeculation, although non-specific, may warrant workup for LV non-compaction cardiomyopathy [25]. The presence of isolated apical LV hypertrophy and/or apical aneurysms may raise the suspicion of apical HCM [5,26,27,28].

### 3.2. Cardiovascular Magnetic Resonance (CMR)

CMR provides a multi-parametric, non-invasive assessment of cardiac volumes, function and tissue characterisation [29]. CMR enables the evaluation of myocardial oedema using T2 (spin-spin relaxation time) short-tau inversion recovery (T2-STIR), T1 (spin-lattice relaxation time) and T2 parametric mapping in patients with suspected CS [1,3,5,20,30,31,32,33]. With a rare exception of “hot phase” ARVC [34], myocardial oedema is not typically present in patients with and therefore, the increased signal on T1/T2-based oedema imaging should raise the possibility of an inflammatory cardiomyopathy [1,5,29].

Late gadolinium enhancement (LGE) imaging is considered the reference standard method for evaluating myocardial fibrosis in non-ischaemic cardiomyopathies [29]. Additionally, CMR has a significant role in assisting the diagnosis of HCM phenotypes when coupled with the clinical picture owing to its ability to tissue characterise the myocardium [35]. Patchy enhancement in areas of focal LV hypertrophy may point to a diagnosis of HCM with focal fibrosis [29], whilst other findings, such as the “Zebra sign”, suggest the presence of cardiac amyloidosis [29]. Conditions such as Anderson–Fabry disease may present with inferolateral mid-wall LGE in the late stages, whilst hypertensive heart disease may have no myocardial fibrosis as an important differential diagnosis [35]. Other less common conditions, such as mitochondrial cytopathies and glycogen storage diseases, can also have mid-wall fibrosis on CMR [35].

Aside from typical features of specific inherited cardiomyopathies, the differentiation between CS and inherited CM based on LGE patterns alone can be challenging since both patient cohorts can have subendocardial, mid-wall, sub-epicardial and/or multi-focal distributions of myocardial fibrosis. Therefore, several additional factors are important to consider when interpreting LGE images of patients suspected of either condition, which may sway the likelihood of the overall diagnosis. For instance, in a patient with an LGE pattern compatible with both CS and inherited CM, the presence of extra-cardiac sarcoidosis (either by biopsy or a high-confidence imaging finding) may increase the likelihood of a CS diagnosis. Further, the additional presence of a positive genetic test in the same scenario, particularly for a compatible inherited CM variant, may support the co-existence of an inherited cardiomyopathy.

### 3.3. FDG-PET-CT

Whole-body FDG-PET-CT is indicated in patients with suspected CS after the performance of CMR [3]. FDG-PET-CT has several important roles in the assessment of CS. Firstly, it provides a qualitative evaluation of the burden and distribution of myocardial inflammation and a quantitative assessment of the intensity of FDG uptake in the form of SUV_max_ [1,32]. Secondly, combining FDG-PET and resting myocardial perfusion imaging helps to identify myocardial scar and inflammation [5,19,20,36,37,38,39,40]. Thirdly, serial FDG-PET-CT scanning enables treatment response assessment and disease progression monitoring. Lastly, whole-body imaging enables assessing the distribution and intensity of extra-cardiac sarcoidosis activity and selecting biopsy targets [1].

Certain features are suggestive of CS, for instance, multi-focal FDG-uptake with perfusion–metabolism mismatch, whilst global intense FDG-uptake may represent a failure of dietary preparation, which is a crucial factor to consider in image interpretation [1,39]. Although the diagnostic performance of FDG-PET-CT is respectable for CS, existing studies tended to be referenced against a clinical diagnosis of CS [39,41]. A negative FDG-PET-CT does not rule out the possibility of CS since it may have been performed during a period of disease remission. The diagnostic role of FDG-PET-CT is less established for inherited CM, although it may be beneficial in patients with HCM, DCM, and Anderson–Fabry disease to assess microvascular dysfunction [5,38]. Importantly, although FDG-PET-CT may help support a diagnosis of CS, it does not distinguish between CS and inherited CM.

### 3.4. CT

Although CT does not directly differentiate between CS and inherited CM, it plays two significant roles in their workup. CT coronary angiogram (CTCA) is now the first-line noninvasive test for assessing stable coronary artery disease (CAD) and helps rule out significant CAD in patients suspected of having non-ischaemic cardiomyopathy [42]. Non-contrast high-resolution CT is also important for assessing pulmonary sarcoidosis, providing an imaging diagnosis and tracking disease progression [1].

### 3.5. Biopsy and Histology

Histologically, sarcoidosis is characterised by the presence of non-caseating granulomas with aggregation of CD68, macrophages, multinucleated giant cells, T lymphocytes, and typical epithelioid cells [1,43]. Although the finding of a positive endomyocardial biopsy (EMB) is considered a definite diagnosis of CS, the sensitivity of EMB is only ~25% from unselected RV biopsies due to the patchy nature of the disease [1,44]. However, this can be increased when cardiac imaging or voltage mapping is used to select biventricular biopsies [1,45,46]. In the presence of a firm clinical diagnosis of CS, EMB is not usually performed, owing to the procedure’s sampling uncertainty and clinical risk to the patients [1,43]. In the case of isolated CS, cardiac biopsy, when available, can help to confirm the diagnosis when the potential risks and caveats of the procedure are considered. Sarcoidosis can sometimes be diagnosed post-mortem or on ex-planted hearts post-cardiac transplantation, either on an incidental or confirmatory basis [8,15,47,48].

In patients with inherited CM, myocardial biopsy may demonstrate certain hallmarks, including myocyte hypertrophy, disarray, and increased myocardial fibrosis commonly seen in HCM [49,50]. In contrast to HCM, DCM has no myocyte disarray, although histology may reveal myocyte hypertrophy and fibrosis [50,51]. The increasing use of advanced cardiac imaging and genetic testing has enabled non-invasive diagnosis of specific inherited CM subtypes; however, histological confirmation remains potentially important in cases where the diagnosis is otherwise inconclusive. New methods incorporating CMR and cardiac biopsy may have a potential role in improving the diagnosis of isolated CS and distinguishing it from other inherited CM and other cardiomyopathies [52]. These novel techniques deserve further clinical validation.

### 3.6. Genetic Mutations

A range of inherited CM has been characterised by specific genetic aetiologies, which have been summarised in the recent 2023 European Society of Cardiology guidelines [5] and the contemporary literature [53]. HCM can be caused by autosomal dominant mutations in sarcomeric proteins [5]. Other autosomal dominant hypertrophic genotypes include those present in TTR amyloidosis and RASopathies [5]. X-linked hypertrophic genotypes include Anderson–Fabry and Danon disease [5]. A number of gene mutations are implicated in DCM and ACM, including LMNA, DES, FLNC, PLN, RBM20 and desmosomal mutations, which tend to be autosomal dominant [5]. Restrictive cardiomyopathies are implicated in mutations in sarcomeric proteins, DES, FLNC, BAG3 and RASopathies [5,54]. Human genetic analysis of cardiomyopathies entails a number of processes, including whole exome sequencing, panel sequencing, cascade screening and variant classification [55,56].

Although there are no specific genetic tests used to diagnose CS, emerging evidence suggests a role in the identification of immune cells with certain molecular signatures within the sarcoid granuloma [57].

### 3.7. Clinical Expertise

Owing to the clinical challenges of CS and inherited CM, the diagnosis and management of these conditions require appropriate clinical specialist expertise rather than being managed in general cardiology clinics [1,5]. For CS, physicians with expertise in CS and cardiac imaging specialists are important in deciding on the diagnosis and the management process [1]. For inherited CM, physicians with inherited CM expertise and those with advanced cardiac imaging with support from geneticists and clinical psychologists are crucial [5,58,59,60]. Additional input is often required from cardiac electrophysiology specialists to advise on and manage arrhythmias and complex cardiac devices. Both conditions require specialist nurse input to provide holistic care to patients. A multi-disciplinary team (MDT)-based approach is key in the diagnosis and management of both conditions.

**Figure 1:**
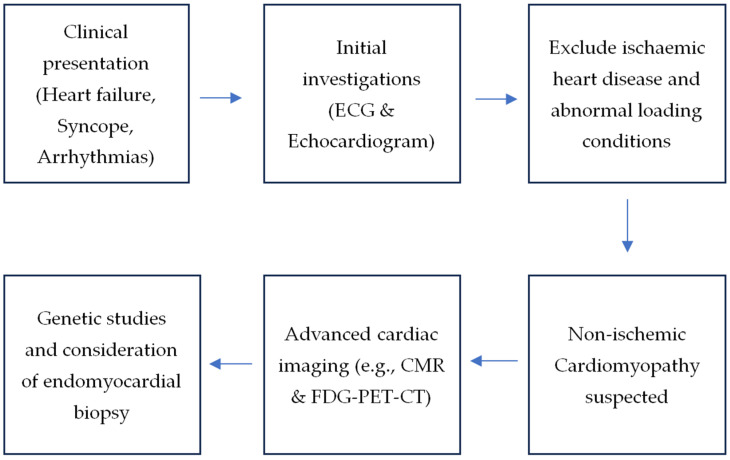
Diagnostic processes for cardiac sarcoidosis and inherited cardiomyopathies.

Figure 1 summarises a possible practical algorithm for the assessment and differentiation between CS and inherited CM. The initial clinical assessment (history taking, clinical examination and ECG) is important in raising the clinical suspicion of CS or inherited CM. This can often be in the context of the age and mode of presentation, e.g., with high-grade AV heart block in a young patient. This clinical suspicion can be followed up with more advanced imaging methods, including echocardiography, CMR and FDG-PET-CT if indicated in the specific clinical scenario. Not all patients require these, and opinions from experts with clinical experience in both conditions are key to guiding the performance of cardiac imaging tests and their interpretation. Based on the cardiac imaging results, further investigations, such as genetic testing or histological assessments, may be warranted. It should be emphasised that there is often no “one-size-fits-all” pathway for differentiating between CS and inherited CM, and the clinical assessment should always be tailor-fitted to the individual patient within the clinical context. It is also important to inform and involve the patient in each step of their care since both conditions if diagnosed, can have psychological impacts. The care provided by an MDT is, therefore, likely the most ideal format.

### 3.8. Artificial Intelligence (AI)

AI-based techniques are expanding and increasing in adequacy of interpretation as more complicated datasets are combined [61]. Trained AI-based ECG models are showing promising results in excluding ARVC in recent studies [61,62] and interpreting CMR in a wide range of cardiovascular diseases, including cardiomyopathies [61,63]. As these models improve and newer methods continue to emerge, we expect an increasing role of AI in the screening and diagnosis of non-ischemic cardiomyopathies. Further work is needed to train AI to differentiate between non-invasive patterns of CS vs. inherited CM, which would be a topic of exciting future research.

**Figure 2:**
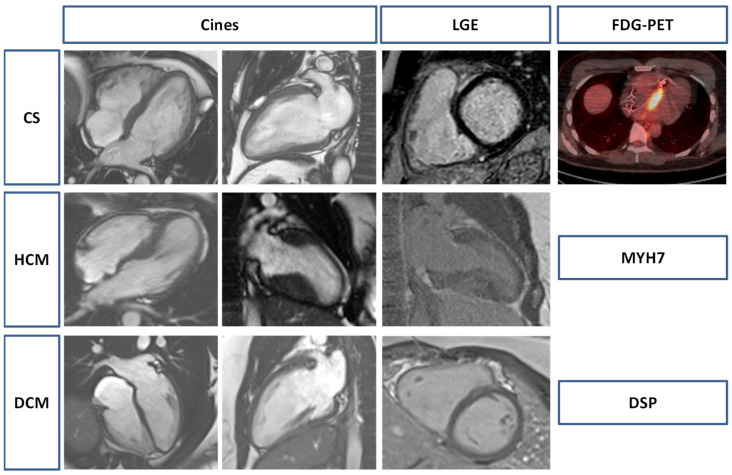
Illustrative imaging of Cardiac Sarcoidosis (CS), hypertrophic cardiomyopathy (HCM) and dilated cardiomyopathy (DCM). The CS patient had LGE in the basal anterior septum on CMR with intense FDG uptake in the septum on FDG-PET. The HCM patient (MYH7 mutation) had asymmetrical left ventricular hypertrophy with patchy fibrosis in the hypertrophied segments. The DCM patient (DSP mutation) had sub-epicardial/mid-wall LGE in the septum and lateral wall. DSP: desmoplakin; FDG-PET: fludeoxyglucose-18 positron emission tomography; LGE: late gadolinium enhancement; MYH7: myosin heavy chain 7.

Illustrative cardiac imaging of CS, HCM, and DCM are shown in Figure 2.

## 4. CS and Hypertrophic Cardiomyopathy (HCM)

A total of 11 case reports (Table 1) documented possible associations between CS and HCM [8,9,10,11,12,13,14,15,16,64]. Patients presented with either syncope, atrial or ventricular arrhythmias or heart failure [8,9,10,11,12,13,14,15,16,64]. In all cases, a diagnosis of HCM was made first, either as a historical pre-existing condition [8,9,11,12,13,14,64] or as the first clinical suspicion based on echocardiography after presentation [10,11,16]. In most cases, a clinical diagnosis of HCM was reached based on the presence of LV septal hypertrophy in the absence of a positive familial history [10,11,13,14,16,64]. Only one patient had a positive genetic variance (heterozygous MYBPC3 variant (p.Arg1268Trp)) [58]. This highlights the need to consider other conditions, such as CS when there is a suspected inherited CM, but the genetic testing is negative.

Five cases had EMB-confirmed CS [10,11,12,13,14]. Although these patients had a prior clinical and imaging diagnosis of HCM, histological evidence did not indicate features of HCM, suggesting that CS had masqueraded as HCM [10,11,12,13,14]. Of the patients without a histological CS diagnosis, a clinical approach was used in three patients to diagnose CS (according to clinical and imaging findings) [8,9,12,14,15]. None of these patients had a genetic or histological diagnosis of HCM beforehand [8,9,12]. It remains possible that these patients had a clinical CS diagnosis masquerading as HCM [8,9,12].

In most cases reviewed, CS was diagnosed many years after the initial HCM diagnosis; during that time, the patient had been managed as an inherited CM rather than a CS [9,12,14]. For example, Yuko et al. reported a case of late CS diagnosis with active myocardial inflammation, which improved with immunosuppressive therapy (corticosteroid), suggesting disease reversibility even with a delayed diagnosis [9]. Agarwal et al. described a 28-year-old lady who was diagnosed with HCM based on clinical presentation and cardiac imaging findings, who progressed to advanced heart failure and died despite mechanical cardiac support [14]. Her deterioration was considered a “burnt-out” phase of HCM, but CS was confirmed on biopsy [14]. This case shows that CS can masquerade as clinical HCM even into the end-stage disease, which deprives the clinical team of the opportunity to offer any CS-specific therapy [14]. The observed septal hypertrophy, in this case, may have been related to myocardial oedema and granuloma [14].

In cases where CS was diagnosed pre-mortem, clinical suspicion for CS only developed after the onset of characteristic complications such as high-grade AVB and/or ventricular arrhythmias [9,11,12,16,64]. These have led to advanced imaging workup and, in some cases, histological confirmation [9,11,12,16,64]. The chronicity between the initial HCM diagnosis and the onset of CS-related complications is important in determining how soon CS can be diagnosed in these patients. In many cases, this time delay is several years, at which point CS may be advanced in disease progression [9,12,14]. Kawakami et al. reported a 76-year-old female who had clinical suspicion for both HCM and CS on CMR, which led to further workup with FDG-PET-CT and cardiac biopsy [10]. Whilst the FDG uptake was localised in the apical LV, suggesting apical HCM, the cardiac biopsy ultimately demonstrated non-caseating granuloma consistent with CS [10]. In patients where advanced cardiac imaging demonstrates features of both HCM and CS, further CS workup can take place sooner, which may reduce any diagnostic delay [10,11,15,16].

In two patients who underwent cardiac transplantation for advanced heart failure after a prolonged HCM diagnosis spanning several years (strong family history, imaging diagnosis, negative genetic studies) [15], their explanted hearts confirmed HCM histologically. However, the pre-transplant CMR raised suspicion of CS in both cases due to marked LGE presence in regions of LVH [15]. These cases highlight the potential perils of using isolated imaging to diagnose CS.

Notably, no cases had both positive genetic tests for HCM and histological confirmation of CS, which would have strongly supported the co-existence of both conditions. However, this combination is also rare in clinical practice owing to the sparing use of myocardial biopsy in both conditions.

## 5. CS and Arrhythmogenic Cardiomyopathy (ACM)

A total of 10 case reports (Table 2) documented a possible association between CS and ACM [8,9,17,47,48,65,66,67]. Most patients were initially diagnosed with ACM before reaching a CS diagnosis [9,17,47,48,65,66]. A diagnosis of ACM was reached based on the presence of left bundle branch block, RV dilatation and dysfunction on echocardiography, with premature ventricular contractions (PVC) [9,17,47,48,65,66]. ECG features such as epsilon waves and T wave inversion in V1-V3 were also incorporated [17,47]. Several patients experienced deterioration, including increased burdens of non-sustained ventricular tachycardia, high-grade AVB, high burdens of PVCs and/or worsening heart failure [9,17,47,65,66]. These features prompted further investigation with advanced cardiac imaging and CT thorax, which demonstrated features of sarcoidosis [9,17,47,66,67]. Histological confirmation of sarcoidosis was found in all patients, with either mediastinal lymph node sampling or on autopsy [8,9,17,47,48,65,66,67].

Ebbinghaus et al. reported a 28-year-old athletic male without a family history of SCD who presented with exercise-related syncope [8]. He had an unremarkable electrocardiogram (ECG) and exercise testing [8]. RV dilatation was found on TTE and CMR [8]. Although he was advised against partaking in sports, he subsequently collapsed with ventricular fibrillation cardiac arrest during sporting activities. He passed away despite resuscitation attempts and ECMO support [8]. The post-mortem samples tested positive for a mutation in plakophilin-2 consistent with ACM [8]. In addition, his autopsy showed extra-cardiac sarcoidosis with no cardiac involvement [8].

In addition to the case reports mentioned above, Philips et al. (Table 2) presented 15 cases of CS, which were initially diagnosed as ARVD/C based on the revised 2010 Task Force Criteria, the common feature among this group is the old age of presentation non-familial pattern of the disease, a greater incidence of heart failure and LV dysfunction in addition to imaging evidence of myocardial fibrosis. Among the 15 patients, histology confirmation of cardiac and extra-cardiac CS was obtained in 10 patients, while the rest had radiological evidence, and all patients responded well to corticosteroid therapy [68].

CMR plays a significant role in the diagnostic workup of both ACM and CS [47,65,67]. However, the differentiation between ACM and CS using CMR alone is challenging. While CS and ACM patients can exhibit LV and RV dilatation and dysfunction, these findings are non-specific for each condition [17,47,67]. Patients with ACM often demonstrate non-ischaemic patterns of LGE in both the LV and RV, which can be associated with local ventricular wall thinning [17,67]. This pattern of myocardial fibrosis can resemble biventricular involvement in specific subtypes of CS [17,47,67]. In these cases, the clinical presentation is often non-specific, including ventricular arrhythmias, which can occur in both disease cohorts [17,47,67].

Further differentiation between ACM and CS often requires additional evidence of sarcoidosis, such as histological confirmation of extra-cardiac sarcoidosis or high-confidence diagnostic imaging of sarcoidosis [8,47,65]. Moreover, a positive genetic test for a compatible variant can help to sway the confidence of an ACM diagnosis and/or dual pathology (ACM and CS) overlap. The multi-faceted consideration of CS and ACM diagnosis differentiation warrants careful evaluation of the clinical data and advanced cardiac imaging findings.

**Table 2:** Diagnosis of ACM and CS in associated cases.

Case Report Author(s)	ACM Diagnosis	CS Diagnosis
Hisaoka et al. [65]	Clinical, ECG, TTE, CMR	CT chest, FDG-PET, Thoracic LN biopsy
Nakamura et al. [69]	Clinical, TTE, negative EMB for sarcoidosis	Clinical, CT chest, Thoracic LN biopsy and steroid responsiveness
Ott et al. [47]	Not diagnosed as ARVC	Clinical, ECG, CT chest, Thoracic LN biopsy and steroid responsiveness
Ott et al. [47]	Clinical, ECG and TTE	Positive autopsy
Ott et al. [47]	Clinical	Positive autopsy
Omotoye et al. [17]	Clinical, TTE, CMR	ECG, FDG-PET, positive EMB, steroid responsiveness
Waki et al. [66]	TTE, CMR	Positive EMB, steroid responsiveness
Siqueiraa et al. [48]	Clinical, TTE, strong family history	Positive EMB of explanted heart
Ebbinghaus et al. [8,67]	Clinical, ECG, positive genetic studies	Extra-cardiac sarcoidosis in autopsy
Chia et al. [67]	Clinical, ECG	CMR, positive EMB, steroid responsiveness
Philips and Madhavan et al. [68]	Total of 15 patients: Clinical, ECG, TTE using (Modified Task Force Criteria)	Clinical, ECG, advanced cardiac imaging 4 had positive endomyocardial biopsies in vivo and explanted heart, 6 had positive extra-cardiac histology, 4 were diagnosed clinically. Steroid responsiveness in all 15 patients

Clinical: clinical presentation of signs and symptoms; ECG: electrocardiography; TTE: transthoracic echocardiography; CMR: cardiovascular magnetic resonance; CT: computed tomography; FDG-PET: ^18^F-fluorodeoxyglucose positron emission tomography; Advanced cardiac imaging: CMR and FDG-PET; EMB: endomyocardial biopsy; thoracic LN biopsy = hilar and/or mediastinal lymph node biopsy.

### CS, ARVC and Ventricular Arrhythmias

The evolution of our understanding of ACM has seen a march from the traditional arrhythmogenic right ventricular cardiomyopathy/dysplasia (ARVC/D) concept, whereby malignant arrhythmias were thought to arise from the RV owing to fibrofatty deposition, to a more contemporary notion of potential LV and/or RV involvement for the arrhythmia foci [70]. In the era of ARVC nomenclature, patients have often been given this diagnosis when presenting with left bundle branch block (LBBB)-type ventricular arrhythmias [71]. Vasaiwala and colleagues found that around 15% of such ARVC patients, in fact, had CS after confirmation by histological biopsy [71]. Further, the authors found that non-invasive diagnostic methods, including CMR and electrophysiological characteristics of the arrhythmia, could not differentiate between CS and ARVC [71].

In patients presenting with ventricular arrhythmias, the diagnostic differentiation between ARVC and CS was particularly challenging in cases of isolated CS, whereby the “clue” from the presence of extra-cardiac sarcoidosis is not available [72]. Patients are, therefore, often labelled as having ARVC until a late stage of clinical progression, for instance, at post-mortem, when a histological diagnosis of CS is made in some cases [72]. Moreover, the task force criteria for diagnosing ARVC have limited efficacy in distinguishing between ARVC and CS [68]. Certain clues, such as high defibrillation thresholds, PR-interval prolongation and high-grade AVB, may assist in raising clinical suspicion for CS when the phenotypical presentation of isolated RV involvement does not differentiate between ARVC and CS [68,73]. Overall, the lack of definitive non-invasive methods to distinguish between CS and ARVC means that clinicians continue to defer to histological biopsies, which invariably lead to diagnostic delays [72]. Such diagnostic delay in the context of patients presenting with potentially compromising ventricular arrhythmias, where treatment decisions need to be made timely and urgently, remains an important clinical problem.

## 6. CS and DCM

Two case reports (Table 3) described patients who were initially diagnosed as DCM with severe LV systolic dysfunction in the absence of significant coronary artery disease [8,14]. These patients underwent advanced cardiac imaging as part of their workup, and subsequently, endomyocardial biopsies took place [8,14]. The patients responded well to immunosuppressive therapy with corticosteroids and heart failure therapy [8,14].

In one case, CMR showed delayed enhancement in a diffuse circumferential pattern, mainly subepicardial, with transmural extension and involvement of the inferior wall of the RV, which would raise suspicion for CS and non-ischaemic cardiomyopathy [14]. Subsequent EMB confirmed CS [14]. In the second case, Ebbinghaus et al. described a patient who presented with high-grade AVB 3 years after initially being diagnosed with a DCM [8]. The patient then developed ventricular arrhythmias ten years after his original presentation [8]. This pattern of presentation suggested the disease progression of CS. Although FDG-PET-CT showed mediastinal lymphadenopathy, it did not demonstrate significant myocardial inflammation, and the EMB showed lymphocytic inflammation without granuloma [8]. The patient experienced clinical improvement after corticosteroid therapy [8]. The delay in the diagnosis of CS was not associated with typical findings of active myocardial inflammation in this case [8].

## 7. Disease Progression

In many cases reviewed, patients are often given a presumed inherited CM diagnosis before a CS diagnosis is made. In some cases, this delay can be several months to years, with the increasing availability of advanced cardiac imaging, such as FDG-PET-CT [74]. It is expected that the suspicion of CS can be raised as early as possible in those patients presenting with cardiac complications. Recent elegant work from the Royal Brompton Hospital showed that early diagnosis of CS in patients presenting with high-grade AV heart block is associated with a better clinical outcome [75]. In patients who are diagnosed with CS, many undergo cardiac device implantations (cardiac resynchronisation therapy CRT), and those who maintain preserved LV systolic function also may have a better long-term prognosis compared to those patients who do not [76]. Remarkably, CS patients who underwent CRT implantations may suffer a worse long-term prognosis compared to ischaemic cardiomyopathy patients despite early LV functional improvement [77]. The comparative outcomes of CS and inherited CM remains an area that deserves further exploration.

## 8. Therapeutic Considerations for CS and Inherited CM

Since complications such as heart failure, ventricular arrhythmias, high-grade AVB and SCD can occur in both CS and inherited CM patients, certain management strategies are shared between the two disease cohorts [1,5]. Both systolic and diastolic heart failure can occur in CS and inherited CM [1,5]. Therefore, guideline-directed medical therapy forms the initial cornerstone of treatment [1,5]. Management of arrhythmias and the use of implantable cardioverter defibrillators (ICD) for SCD prevention have been detailed in recent guidelines for both conditions [1,5]. The endpoint therapy of cardiac transplantation is available to patients with CS or inherited CM [1,5].

Treating myocardial inflammation with immunosuppressive drugs is beneficial prognostically and regarding scar prevention and arrhythmia management [1,5]. Although delayed suspicion of CS (masquerading as inherited CM) may be coupled with delays in identifying myocardial inflammation, this relationship is not proven. Indeed, some reports indicate that despite several years of delay in CS diagnosis, myocardial inflammation found on FDG-PET-CT could still be reversed with corticosteroid therapy [9,11,47,64,66,67]. Although the use of immunosuppression is not common in inherited CM, new evidence is emerging regarding the role of inflammation in ACM, which potentially heralds the possibility of immunosuppressive and/or immunomodulatory therapies in the future [78].

In patients presenting with sustained ventricular arrhythmias refractory to medical therapy, the distinction between CS and inherited CM has important clinical implications [5,79,80,81,82,83]. Whilst ventricular tachycardia (VT) ablation is indicated in inherited CM patients presenting with refractory sustained VT, immunosuppression has an additional role in CS patients [5,82,83]. This is further complicated by the observation that VT ablations in CS patients, though they can be effective, may have a high VT recurrence rate [81]. From a practical viewpoint, it may not be possible to perform FDG-PET-CT studies to guide immunosuppressive therapy in CS patients presenting with refractory VT or VT storms due to concerns over clinical instability. Indicators from ECG, CMR or electro-anatomical mapping of septal involvement may raise suspicion of CS in patients with left bundle branch block morphology VT [79,80], which should be taken into account in the overall clinical management. The differentiation between inherited CM and CS remains challenging in patients presenting acutely with VT, which deserves further investigation.

The genetic predisposition of certain mutations, such as desmoplakin, is well established in inherited CM, whereby the cascade screening of first-degree relatives of the proband is critical to their comprehensive management [5]. The genetic predisposition of CS patients is much less well-known. Currently, no genetic tests are available in the clinical arena to assess and screen CS patients and their relatives. Therefore, genetic testing is an important management strategy that sets the two disease cohorts apart. Most of the cases reviewed showed a propensity for an inherited CM to be diagnosed first, providing an opportunity for appropriate genetic screening. In practice, regardless of the later diagnosis of CS, inherited CM management can be appropriately and timely instigated upon a positive genetic diagnosis.

Emerging genetic therapy methods represent another important future advance in the management of inherited CM patients. These include CRISPR-Cas9 DNA Base-Editing, Prime-Editing, and other therapies [84]. The clinical relevance of these novel therapies is likely to be evaluated further in the future.

## 9. Conclusions

The differentiation between CS and inherited CM is a significant clinical problem due to their similar clinical presentations and diagnostic imaging findings. Patients are often diagnosed with an inherited CM first, e.g., HCM, ACM or DCM, and undergo treatment accordingly. Often years later, clinical suspicion for CS is raised when a patient presents with complications, such as high-grade AVB or ventricular arrhythmias, which leads to a workup for CS. In many cases, CS had masqueraded as an inherited CM, leading to diagnostic delays and instigation of treatment at later stages of disease progression. The differentiation between CS and inherited CM requires careful evaluation of the clinical information and advanced cardiac imaging data. Cardiac biopsies can provide a definitive diagnosis with the caveat of being invasive and prone to sampling errors. Genetic testing and affirming extra-cardiac sarcoidosis diagnoses can aid the overall diagnostic confidence of inherited CM and CS, respectively. CS and other potential mimics should be considered in the absence of positive genetic studies for inherited CM.

## Figures and Tables

**Table 1:** Diagnosis of HCM and CS in associated cases.

Case Report Author(s)	HCM Diagnosis	CS Diagnosis
Agarwal et al. [14]	Clinical, ECG, TTE	Clinical, CT Chest, Advanced cardiac imaging, positive EMB
Miki et al. [9]	Clinical, TTE	ECG, TTE, Advanced cardiac imaging, steroid responsiveness
Kawakami et al. [10]	Clinical, TTE, Advanced cardiac imaging	Positive EMB
Feldman et al. [11]	Clinical, TTE, CMR	ECG, Advanced cardiac Imaging, positive EMB, steroid responsiveness
Haley et al. [16]	Clinical, TTE	ECG, Advanced cardiac imaging, Thoracic LN biopsy
Pham et al. [64]	Clinical, TTE	ECG, Advanced cardiac imaging, positive EMB, steroid responsiveness
Mathias et al. [13]	Clinical, TTE, CMR	FDG-PET, positive EMB
Saitou et al. [12]	Clinical, TTE	ECG, Advanced cardiac imaging, inconclusive EMB
Ebbinghaus et al. [8]	Clinical, ECG, positive genetic variance of unknown significance (heterozygous MYBPC3 variant (p.Arg1268Trp))	Advanced imaging, steroid responsiveness, negative EMB
Sellers et al. [15]	Clinical, Echo, positive EMB on explanted heart	Clinical, Advanced imaging, clinical
Sellers et al. [15]	Clinical, Family history, positive EMB on explanted heart	Clinical, Advanced imaging, clinical

Clinical: clinical presentation of signs and symptoms; ECG: electrocardiography; TTE: transthoracic echocardiography; CMR: cardiovascular magnetic resonance; CT: computed tomography; FDG-PET: ^18^F-fluorodeoxyglucose positron emission tomography; Advanced cardiac imaging: CMR and FDG-PET; EMB: endomyocardial biopsy; thoracic LN biopsy = hilar and/or mediastinal lymph node biopsy.

**Table 3:** Diagnosis of DCM and CS in associated cases.

Case Report Author(s)	DCM Diagnosis	CS Diagnosis
Agarwal et al. [14]	Clinical, TTE, ECG No family history or genetic studies	CMR, positive EMB and steroid responsiveness
Ebbinghaus et al. [8]	Clinical, Imaging, Positive family history No genetic studies	Initially negative EMB Delayed diagnosis after ten years. Clinical (high degree AV block), advanced cardiac imaging, and positive EMB.

Clinical: clinical presentation of signs and symptoms; ECG: electrocardiography; TTE: transthoracic echocardiography; CMR: cardiovascular magnetic resonance; FDG-PET: ^18^F-fluorodeoxyglucose positron emission tomography; Advanced cardiac imaging: CMR and FDG-PET; EMB: endomyocardial biopsy.
